# Factors influencing international and U.S. dentists’ interest in advanced periodontal education: a pilot study

**DOI:** 10.1186/s12903-021-01728-4

**Published:** 2021-07-21

**Authors:** Andre Paes B. da Silva, Hazem Saqqal, Andrew Guirguis, Uma M. Irfan

**Affiliations:** 1 CWRU Health Education Campus, Dental Clinic / 2nd Fl – 240, 9601 Chester Avenue, Cleveland, OH 44106 USA; 2 Present Address: Associates in Periodontics, 1775 Williston Rd, South Burlington, VT 05403 USA; 3 Private Dental Practice, 435 tutus point #1011, FL Oviedo, USA; 4 School of Dental Medicine, CWRU, 2124 Cornell Rd, Cleveland, OH 44106 USA

**Keywords:** Dental residency, Graduate education, Periodontal education

## Abstract

**Background:**

The enrollment of international periodontal students in U.S. dental schools has been increasing in recent years. Interest in applying to a periodontics specialty program may differ between U.S and international dental school graduates. The purpose of this study is to assess, from the perspective of periodontal residents, (1) factors that interest dental students to apply to periodontics programs and (2) differences in background and interest between U.S and international graduates.

**Methods:**

A 20-question survey was sent out electronically to periodontics residents. The survey questions were designed to obtain information on the participants’ backgrounds, factors that influenced them to specialize in periodontics, and their preferred features of graduate periodontics programs. The data were analyzed using descriptive statistics for socio-demographic variables, a Wilcoxon two sample test to compare mean Likert scale scores, and Fisher’s exact test for associations between comparison groups.

**Results:**

Of the two hundred residents invited to participate, 28% responded. The majority of the respondents stated that interest in implantology, previous exposure to periodontal procedures, interest in improving periodontal surgery skills, a good relationship with periodontics faculty, the residency curriculum, advanced program and faculty reputation as influencing factors in selecting periodontics as specialization. The majority of international graduates have up to $50,000 dollars in student debt; by comparison, half of the domestic graduates have a debt of over $250,000 dollars (*p* ≤ 0.05). Working experience as a dentist was significantly greater among international residents (73%) in comparison to U.S graduates (32%). In contrast with international graduates, U.S graduates more frequent reported that good relationships with the periodontics predoctoral faculty contributed to their interest in periodontics (*p* ≤ 0.05). Program cost and location had a greater impact on the decision of U.S. graduates than international graduates (*p* ≤ 0.05).

**Conclusions:**

Overall, factors associated with personal finance and predoctoral education have a greater impact on the decision of American graduates than international graduates to pursue an advanced education in periodontics, which may influence the increased enrollment of international students.

## Background

Fifty-five university- and hospital-based graduate periodontics programs exist in the United States. Forty-five programs admit international students who do not have a U.S dental license. According to the American Dental Association (ADA)’s annual Survey of Advanced Dental Education and the Council on Dental Education and Licensure (CDEL), the number of enrolled international periodontal students in U.S. dental schools increased from 2012 to 2018. In the graduating class of 2012, 27% of graduates from advanced programs in periodontics were international students, compared to 33% in 2020. Moreover, the number of programs accepting international students increased from 39 programs (2012) to 47 programs (2020). However, the percentage of international students who enrolled in other specialties such as endodontics and prosthodontics has remained almost stable [1].

In our previous study, from the periodontics department chairpersons’ perspectives reported by Luke Hearty et al., specialty clinic rotations and elective courses appear to increase student interest in a periodontal residency program. Periodontal residencies that offer externships had a greater number of candidates. However, factors such as residency stipends and fellowships did not have great influence on dentist interest in a periodontal program [2]. Data from a survey on graduating dental students, suggested that mentoring influenced students interest in residencies, indicating the significance of exposure to the advanced program educators and residents [3]. Nevertheless, in another study in the U.S., it was reported that graduating dental students with a debt of at least $100,000 were more likely to start practicing dentistry after graduation than students with lower debt, even after adjusting for the impact of individuals who were influential on students’ career decisions [4].

Differences between dental students in terms of social, economic and cultural backgrounds are expected to influence their career plans. Surveyed dental students from different Middle Eastern nationalities in a Jordan Dental school agreed on most factors affecting their choice of a specialty, except for the reputation of the specialty [5]. Authors of a student survey conducted in one dental school in U.K. reported that “having a talent in the field” had the largest positive influence on pursuing a specialist career [6]. Saudi Arabia dental students reported that the influence of family members in the dental profession, and specific interest in patient population as significant factors in choosing a specialty. Other important factors were variety of non-clinical duties, and research opportunities [7].

Interest in applying to a periodontics specialty program may differ between U.S and international dental school graduates. The aim of this study is to assess the perspective of periodontal residents regarding (1) factors that attract dental students to apply to periodontics programs and (2) differences in background and interest between U.S and international graduates.

## Methods

### Participants

The research protocol for this study was approved by the Case Western Reserve University Institutional Review Board as exempt from oversight (IRB-2018-1242). The survey was carried out in accordance with relevant guidelines and regulations. In 2018, a 20-question survey (Table 1) was sent out electronically to periodontics graduate program residents. The survey was placed on the Surveymonkey website and the link to participate in the survey was sent out to 200 residents, who were student members of the American Academy of Periodontology (AAP) and were enrolled in a residency program. The survey contained a cover letter explaining its purpose, how the data would be used, stated that the data would be anonymous. The request to participate in the survey was sent three times by e-mail in 30-day intervals. Respondents completed the survey anonymously and voluntarily.

**Table 1:** Survey questionnaire

Please answer all questions to the best of your knowledge and ability
Respondent Id:
1. Age in years:
2. Gender: 1 = Male; 2 = Female
3. Are you a graduate from an International dental school? 1 = Yes; 0 = No
4. How much educational loan/debt did you have prior to starting your residency in Periodontics? (a) None (b) $50,000—$150,000 (c) $150,000—$250,000 (d) More than $250,000
5. Have you practiced dentistry before applying to a Periodontics Advanced Training Program?
6. If yes, for how long have you practiced dentistry?
7. Did you experience any of the following before attending advanced periodontal education program? You may choose more than one choice a. Working as general dentist b. Advanced training in general dentistry c. Master’s degree or PhD. In related field d. Research e. None of the above
8. Have you done an externship/Fellowship in a Periodontics department prior to applying to the Advanced Periodontal Education Program?
Please respond to the statements below that represent preferred graduate program features associated with choosing advanced periodontal education; with a score on a scale of 1 to 5, [1 = Strongly disagree; 2 = Disagree; 3 = Neutral; 4 = Agree; and 5 = Strongly Agree] 9. I am familiar with the curriculum contents of this particular program that you are in currently 10. The program and faculty member reputation was important to me 11. Cost of attending the program was a significant factor to be considered for me 12. Location of the program counted for me 13. I look forward to Research opportunities in the program 14. I have special interest in Implantology 15. I had a good relationship with periodontal faculty while attending dental school 16. I want to improve my capabilities in performing periodontal surgeries 17. Advanced Periodontal education is one of the ways to obtain a license to work in USA 18. The exposure to periodontal procedures had built my interest in periodontics 19. I was interested in the Periodontology course in my dental school
20. What are your future plans after graduation? You may choose more than one choice a. Staying in the US b. Try for an Academic full time position c. Join a private practice d. Try for an Academic part-time position e. Apply for further education f. Practice in home country (International graduates only)

### Study questionnaire

The survey included questions that were dichotomous, had multiple choices, or had a 5-point Likert scale (1 = strongly disagree to 5 = strongly agree). Some of the standardized survey questions were derived from previous investigations [3, 8], and others were created to address research objectives. The modified survey questionnaire was pilot tested among colleagues of the authors to validate questions before it was uploaded online.

The survey questions were framed to obtain information related to the participants’ socio-demographic characteristics, factors in selecting periodontics as an area of specialization (interest in periodontal surgeries, interest in implantology, previous exposure to periodontal surgeries, previous positive interactions with periodontal faculty, periodontology courses in dental school, and previous externship/fellowship), and the preferred graduate periodontics program features (location, cost, curriculum, faculty reputation, and research opportunities). Previous experience before enrolment in the periodontics program and goals after completion of the program was also surveyed.

### Statistical analysis

The data were analyzed using IBM SPSS software version 27, with descriptive statistics on socio-demographic variables; Chi-square analysis and/or Fisher’s exact test was performed to test associations between socio-demographic variables and previous experiences in dentistry and research among International and US graduates; and a non-parametric Wilcoxon two sample test was done to compare mean Likert scale scores on factors influencing advanced periodontal education and future prospects among US and International graduates. The statistical significance for all tests was set at *p* < 0.05.

## Results

A total of 58 (28%) invited residents responded to the survey. About 50% of respondents were between 30 and 40 years of age and had less than $50,000 in educational debts prior to their attendance in the residency program, and 78% were male with 65% having graduated from an accredited dental school in the U.S (Table 2).

**Table 2:** Distribution of the Socio-demographic characteristics of International versus US Graduates

Socio-demographic variables	Dental graduates			*p*-value
	All N = 58 (100%)	International N = 20 (35%)	U.S N = 38 (65%)	
Age in years				
< 30	18 (31.0)	2 (10.0)	16 (42.1)	0.0163*
30–35	26 (44.8)	12 (60.0)	14 (36.8)	
36–40	2 (3.5)	2 (10.0)	0 (0)	
> 40	12 (20.7)	4 (20.0)	8 (21.1)	
Gender				
Female	13 (22.4)	5 (25.0)	8 (21.1)	0.4876
Male	45 (77.6)	15 (75.0)	30 (78.9)	
Educational debt (US dollars) prior to residency in Periodontics: None	25 (43.1)	14 (70.0)	11 (28.9)	0.0053*
< 50,000	5 (8.6)	2 (10.0)	3 (7.9)	
50,000150,000	7(12.1)	2 (10.0)	5 (13.2)	
150,000–250,000	3 (5.2)	1 (5.0)	2 (5.3)	
> 250,000	18 (31.0)	1 (5.0)	17 (44.7)	

Chi-square/Fisher’s exact test

^*^Significant at *p* < 0.05

The majority of international graduates’ (60%) were between the ages of 30 and 35 years that was significantly (*p* ≤ 0.05) different with only 37% of the U.S graduates in this age category. The majority of the international graduates (70%) had no educational debt and in comparison, it was significantly different (*p* ≤ 0.05), with approximately 21% of US graduates had incurred an educational debt less than $150,000, and 50% had a debt greater than $150,000 dollars (Table 2).

The distribution of respondents’ dental-associated experience prior to the periodontics residency included 19% with a Master’s or PhD training, 24% with research experience, 22% with advanced training in general dentistry, 38% with an externship/fellowship, and approximately 45% had prior experience practicing dentistry. The response rates for working as a general dentist prior to residency enrollment was significantly (*p* ≤ 0.05) different between international (70%) and U.S. graduates (32%) (Table 3).

**Table 3:** Comparison between US and International Residents’ Experience in dentistry and research prior to periodontal residency program

Experience in dentistry / research	Dental Graduates			*p* value
	All N = 58 (100%)	International N = 20 (35%)	U.S N = 38 (65%)	
Worked as general dentist				
Yes	26 (44.8)	14 (70.0)	12 (31.6)	0.0056*
No	32 (55.2)	6 (30.0)	26 (68.4)	
Advanced training in GeneralDentistry:				
Yes	13 (22.4)	6 (30.0)	7 (18.4)	0.2473
No	45 (77.6)	14 (70.0)	31 (81.6)	
Master’s Degree / PhD. training in related field				
Yes	11 (18.9)	5 (25.0)	6 (15.8)	0.3037
No	47 (81.1)	15 (75.0)	32 (84.2)	
Experience in research				
Yes	14 (24.1)	6 (30.0)	8 (21.1)	0.3274
No	44 (75.9)	14 (70.0)	30 (78.9)	
Experience in externship/fellowship in a periodontics department prior to periodontics residency enrolment				
Yes	22 (37.9)	9 (45.0)	13 (34.2)	0.2999
No	36 (62.1)	11 (55.0)	25 (65.8)	
No previous experience in dentistry / research				
Yes	13 (22.4)	0 (0)	13 (34.2)	0.0017*
No	45 (77.6)	20 (100)	25 (65.8)	

Chi-square/Fisher’s Exact Test

^*^Significant at *p* < 0.05

Survey responders answered questions on factors that may influence the selection of periodontics as an area of specialization. The majority of the respondents stated that interest in implantology, previous exposure to periodontal procedures, interest in improving periodontal surgery skills, and a good relationship with periodontics faculty as influencing factors in selecting periodontics as an area of specialization (Table 4). International students were significantly less frequent than U.S. graduates to report that the reason they chose the field of periodontics was due to a positive relationship with a periodontics faculty member (*p* ≤ 0.05). Moreover, unlike U.S. graduates, international graduates reported that the completion of a periodontics program was one way to obtain a dental license to practice in the U.S.; this influenced their interest in periodontics (*p* ≤ 0.05) (Table 4).

**Table 4:** Distribution of mean Likert Scale scores on factors influencing the choice of Periodontics as a Specialty Career among US and International Graduates

Influencing factors for choice of periodontics as specialty career	All graduates (n*)	US Graduates Mean Score ± sd (n*)	International graduates Mean Score ± SD (n*)	*p* value
Interest in Implantology	4.06 ± 1.27	4.00 ± 1.23	4.17 ± 1.38	0.3226
Good Relationship with Periodontal Faculty	3.49 ± 1.66	3.89 ± 1.41	2.38 ± 1.85	0.0149*
To improve Periodontal Surgery capabilities	4.80 ± 0.62	4.75 ± 0.73	4.89 ± 0.32	0.5243
One way to obtain License to work in the US	2.12 ± 1.56	1.38 ± 0.96	2.92 ± 1.73	0.0088*
Exposure to Periodontal Work has increased interest in the subject	4.50 ± 1.07	4.47 ± 1.05	4.56 ± 1.15	0.4977
Periodontal course in my school	3.06 ± 1.59	3.27 ± 1.48	2.63 ± 1.78	0.2242

Wilcoxon Two-Sample Test

^*^Significant at *p* < 0.05. (n*) Number of respondents vary due to non-response

The majority of the respondents agreed on the importance of the influencing factors associated with the program characteristics, such as the curriculum, the program and faculty reputation. However, in selecting a residency, cost and location had a greater impact on the decision of U.S. graduates than international graduates (*p* ≤ 0.05) (Table 5).

**Table 5:** Distribution of Mean Likert Scale scores of Periodontal residents’ responses on influencing factors for choosing Periodontal programs

Influencing factors for choosing periodontal programs	All Graduates (n*)	US Graduates Mean Score ± sd (n*)	International graduates mean score ± SD (n*)	*p *value
Curriculum contents	3.91 ± 1.06	3.91 ± 1.06	4.05 ± 1.14	0.4802
Program and Faculty Member Reputation	3.93 ± 1.27	3.83 ± 1.34	4.11 ± 1.13	0.4464
Cost of Attendance	3.90 ± 1.36	4.23 ± 1.14	3.13 ± 1.55	0.0131*
Location of the Program	3.24 ± 1.63	3.56 ± 1.59	2.53 ± 1.50	0.0322*
Research Opportunities of the Program	2.64 ± 1.51	2.44 ± 1.56	3.07 ± 1.33	0.1612

Wilcoxon two-sample test

^*^Significant at *p* < 0.05

(n*) Number of respondents vary due to non-response

U.S. and international graduates had similar interests pertaining to their future plans. Yet, international graduates were more interested in holding a part-time (40%) academic position than were U.S. graduates (15%) (*p* ≤ 0.05). One-third of the international graduates reported that they intend to practice dentistry in their home country (Table 6).

**Table 6:** Distribution of reported choices for Future Career Plans of US Dental Graduates versus International Dental Graduates

Reported future plans of dental graduates	All graduates (n = 58)		US dental graduates (n = 38)		International dental graduates (n = 20)		*p*-value
	Yes n (%)	No n (%)	Yes n (%)	No n (%)	Yes n (%)	No n (%)	
Staying in the US	30 (51.7)	28 (48.3)	22 (57.9)	16 (42.1)	8 (40.0)	12 (60.0)	0.1539
Full time Academic Position	8 (13.8)	50 (86.2)	4 (10.5)	34 (89.5)	4 (20.0)	16 (80.0)	0.2706
Private Practice	42 (72.4)	16 (27.6)	27 (71.1)	11 (28.9)	15 (75.0)	5 (25.0)	0.5017
Part time Academic Position	14 (24.1)	44 (75.9)	6 (15.8)	32 (84.2)	8 (40.0)	12 (60.0)	0.0441*
Further Education	6 (10.3)	52 (89.7)	3 (7.9)	35 (92.1)	3 (15.0)	17 (85.0)	0.3372
Practice in Home Country	7 (12.1)	51 (87.9)	1 (2.6)	37 (97.4)	6 (30.0)	14 (70.0)	0.0052*

Wilcoxon two-sample test

^*^Significant at *p* < 0.05

## Discussion

In the present study, from the perspective of periodontics residents, interest in undertaking a graduate periodontics program is shared among U.S and international graduates. Overall, the majority of respondents stated that interest in implantology, previous exposure to periodontal procedures, interest in improving periodontal surgery skills, a good relationship with periodontics faculty, the residency curriculum, advanced program features and faculty reputation as factors that attract dental graduates in selecting an advanced periodontics program. One of the top influential factors was previous exposure to periodontal procedures. This result is in agreement with our previous study, which was reported in the opinion of periodontics department chairmen that specialty clinic rotations and elective courses increase student interest in applying to advanced periodontal education [2]. However, there were differences between U.S. and international graduates in terms of background and factors that influenced them to commit to further education in periodontics, such as debt, dental work experience, a good relationship with predoctoral faculty, advance training cost, and location.

In the U.S., the average educational debt for a dental student was $292,000 in 2019 [9]. This is in agreement with the present study, where a significantly higher proportion of U.S. dental graduates, had educational debts greater than $150,000 as compared to international graduates. According to the present survey, in comparison to American graduates, international graduates tend to be older and have more work experience as a general dentist prior to attending a periodontics program, which may contribute to their having less debt. Furthermore, international graduates frequently have financial support from their home country’s government for higher education. In agreement with the present study, student debt [3, 4, 8, 10], program cost [11] and location [12, 13] have been reported as significant factors that influenced the students’ decision to specialize in dentistry. In the U.S., the average income for general dentists is lower than that of dental specialists [14]. However, American students may be drawn to this career choice by the ability to enter the workforce earlier. Interestingly, in our previous study, from the perspective of program chairmen, the offering of a stipend does not affect the number of applications to the periodontics programs [2]. Perhaps the increasing numbers of international students in dental higher education over time may be due to the fewer financial responsibilities compared to the U.S. graduates’ financial burden.

In the present study, in comparison to international graduates, U.S. graduates reported that a good relationship with the pre-doctoral faculty had a positive impact on developing interest in a periodontics program. This result ties with a previous study wherein it was found that strong faculty student interaction tended to foster interest in a career in periodontics [15]. In agreement, prosthodontics residents reported that advice from predoctoral mentors was an important influential factor in choosing prosthodontics as a career [16]. However, in a Saudi Arabian study, students reported that the influence of family members in the dental profession are of high importance in a selection of a specialty [7]. This variance in opinions among international and American graduates may be influenced by differences in cultural backgrounds. For example, it has been reported that prestige is an important influential factor on choosing a specialty for Turkish, Saudi and Iranian dental graduates. However, it is not an essential factor for western dental graduates, such as British and Danish [6, 17–19]. Interestingly, in the present study, international graduates reported that completion of a periodontics program was one way to obtain a dental license to practice in the United States. Also, approximately 30% of international graduates had plans to practice in the United States. Completion of a CODA accredited specialty program is one of the three educational pathways in order to be eligible for licensing in the U.S. [20]. Perhaps, given the decreased amount of debt, international graduates may have more disposable revenue than domestic graduates, and may consider dental specialization in the U.S. as a financial opportunity to earn a higher income as a specialist.

There were limitations to this study. Not all residents in all U.S. programs could be reached, since their contact information was not accessible through the AAP directory website, which is meant to have residents’ contact information. The low response rate (28%) could be partially explained by residents’ main focus on completion of their residency program, by the workload needed to successfully fulfill all of a program’s requirements, and by a potentially outdated list of e-mail addresses in the AAP directory. However, given the small sample, this study identified interesting and informative factors that describe the choice differences between U.S. and international graduates in selecting periodontal programs. A post-hoc power analysis revealed power ranging from 53.3 to 95.8% for dichotomous variables and 65.8% to 80.5% for continuous variables that were analyzed to detect differences between US and International graduates in this study. Studies with small sample size could potentially reveal important characteristics that could be expanded with larger studies and may be included in systematic reviews [21, 22]. Further studies with larger samples are required to increase the power to detect and better understand the differences in influencing factors between U.S. and international graduates’ interests in advanced periodontal education.

## Conclusion

This study found that a good relationship with pre-doctoral faculty had a greater influence on American graduates than international graduates when choosing periodontics as a career. Personal finances and program location had a greater negative impact on American graduates than international graduates when selecting an advanced periodontics program of choice. Hence, the increasing cost of dental education in the U.S. as it translates to educational debt incurred by US graduates; could explain the increased enrollment of international students in advanced periodontal education.

## Data Availability

The datasets used and/or analyzed during the current study are available from the corresponding author on reasonable request. "The dataset(s) supporting the conclusions of this article is(are) included within the article.
